# An Efficient Retinal Fluid Segmentation Network Based on Large Receptive Field Context Capture for Optical Coherence Tomography Images

**DOI:** 10.3390/e27010060

**Published:** 2025-01-11

**Authors:** Hang Qi, Weijiang Wang, Hua Dang, Yueyang Chen, Minli Jia, Xiaohua Wang

**Affiliations:** 1 School of Integrated Circuits and Electronics, Beijing Institute of Technology, Beijing 100081, China; qihang@bit.edu.cn (H.Q.); wangweijiang@bit.edu.cn (W.W.); danghuabit@163.com (H.D.); yyc@bit.edu.cn (Y.C.); 7420220059@bit.edu.cn (M.J.); 2 BIT Chongqing Institute of Microelectronics and Microsystems, Chongqing 401332, China

**Keywords:** optical coherence tomography, large kernel attention, multi-scale perception, lightweight, retinal fluid segmentation, deep learning

## Abstract

Optical Coherence Tomography (OCT) is a crucial imaging modality for diagnosing and monitoring retinal diseases. However, the accurate segmentation of fluid regions and lesions remains challenging due to noise, low contrast, and blurred edges in OCT images. Although feature modeling with wide or global receptive fields offers a feasible solution, it typically leads to significant computational overhead. To address these challenges, we propose LKMU-Lite, a lightweight U-shaped segmentation method tailored for retinal fluid segmentation. LKMU-Lite integrates a Decoupled Large Kernel Attention (DLKA) module that captures both local patterns and long-range dependencies, thereby enhancing feature representation. Additionally, it incorporates a Multi-scale Group Perception (MSGP) module that employs Dilated Convolutions with varying receptive field scales to effectively predict lesions of different shapes and sizes. Furthermore, a novel Aggregating-Shift decoder is proposed, reducing model complexity while preserving feature integrity. With only 1.02 million parameters and a computational complexity of 3.82 G FLOPs, LKMU-Lite achieves state-of-the-art performance across multiple metrics on the ICF and RETOUCH datasets, demonstrating both its efficiency and generalizability compared to existing methods.

## 1. Introduction

Optical Coherence Tomography (OCT) is a non-invasive imaging modality that provides high-resolution cross-sectional views of retinal structures at a microscopic scale [1]. OCT utilizes a broadband light source that is split into two paths: one beam is directed at the sample to detect backscattered light, while the other is sent to a reference system. Through low-coherence interference, signals with an optical path difference within the coherence length are detected, allowing depth information to be extracted and enabling the generation of 2D (B-scan) or 3D structural images [2]. This exceptional capability to visualize the retina’s intricate layer-wise architecture has established OCT as an indispensable tool in ophthalmology, particularly for the diagnosis and monitoring of conditions such as Macular Edema [3] and other retinal pathologies. The accurate segmentation of OCT images is crucial for identifying fluid accumulations, lesions, and structural abnormalities, as quantitative analysis of abnormal fluid aids in formulating targeted treatment plans. Recent advancements in Artificial Intelligence (AI)-driven automatic segmentation have transformed diagnostic support systems, providing robust and adaptable solutions tailored to diverse clinical scenarios [4,5,6,7,8]. By predicting the majority of fluid regions, algorithms can significantly reduce clinicians’ workload and minimize the risk of misdiagnosis, thereby enabling more efficient and reliable diagnostic interventions.

The Fully Convolutional Network (FCN) [9] is widely regarded as the foundational work in pixel-level dense prediction, playing a vital role in driving the rapid advancement of intelligent segmentation technology. As a significant evolution, UNet [10] introduces a U-shaped encoder–decoder architecture with skip connections, delivering exceptional performance and establishing itself as the de facto standard for medical image segmentation [11]. UNet implements a process analogous to information transmission, as described in information theory, through its symmetric encoder–decoder structure. In this process, the encoder compresses the input image by extracting features, while the decoder restores the image resolution to its original size to reconstruct the predicted information. The skip connections serve as an additional pathway in the information transmission process, helping to retain more low-level semantic information. This structure effectively reduces potential information loss during transmission. The U-shaped architecture has inspired a series of variants, including UNet++ [12], Att-UNet [13], and others. However, the principle of OCT imaging introduces significant challenges to lesion edge detection, as it results in high noise intensity, low contrast, and blurred edge structures. Consequently, directly extracting the edge information becomes difficult. Additionally, the varying shapes and sizes of fluid regions further complicate the segmentation task.

A series of measures have been proposed to improve the segmentation performance of OCT images. For example, Lu et al. [14] utilize graph cuts for preprocessing and apply random forest classifiers to eliminate incorrectly labeled liquid areas. RetiFluidNet [15] introduces self-adaptive attention and deep supervision strategies to enable hierarchical learning, while Rahil et al. [16] employ deep ensemble learning architectures to enhance multi-category segmentation. However, these methods are mainly based on U-shaped architectures with fixed-size convolution kernels and do not fully explore the potential of wide-range receptive fields. Blurred edge regions often exhibit subtle gradient changes, making them challenging to accurately identify using a single narrow-view convolution kernel (e.g., 3×3 kernel [17]). Effective boundary segmentation often necessitates the capability to model wide-range dependencies, as boundary cues typically rely on broader contextual information. This process integrates data from neighboring regions, enhancing the model’s ability to infer potential boundary locations. Expanding the receptive field, as demonstrated by the use of Dilated Convolution [18] in DeepLab [19] and the multi-scale receptive field integration in PSPNet [20], is a widely adopted and effective strategy in computer vision tasks. However, despite significant advancements, the inherent local inductive bias of Convolutional Neural Networks (CNNs) can result in performance bottlenecks, as CNNs are less effective at capturing global long-range dependencies [21]. These global contextual dependencies are crucial for understanding correlations between scattered but related regions in an image, such as dispersed retinal fluid regions.

Recently, global modeling approaches have emerged as promising solutions to overcome the limitations of CNNs. Among these, Vision Transformer (ViT) [22], derived from the self-attention mechanism [23] originally developed in Natural Language Processing (NLP), has gained significant attention. While this approach effectively captures the global receptive field, it incurs significant computational costs due to its quadratic complexity [24] and high parameter requirements. This limitation restricts the model’s training efficiency on small-scale datasets, particularly in medical application scenarios. Conversely, capturing local patterns remains essential, as it corresponds to the local edge and texture information in OCT images. However, Transformers demonstrate inherent limitations in this capability [25]. Therefore, some approaches aim to design CNN–Transformer hybrid models [26,27,28] to balance structural information and long-range relationships while optimizing computational efficiency. In the context of customized models for OCT tasks, Cao et al. [29] propose a vertical self-attention module at the bottleneck of the U-shaped network, tailored to the characteristics of retinal cross-sectional images. FNeXter [30] combines ConvNeXt [31] and Transformer architectures to capture both local details and global features. Furthermore, large kernel approaches [32,33,34] have garnered attention, as they are regarded as effective methods for balancing global and local feature representation. Although considerable effort has been devoted to developing resource-efficient and accurate methods, these approaches generally incur higher overhead compared to naive CNN-based models, making them less suitable for devices with limited computing resources, particularly mobile auxiliary diagnostic devices.

Motivated by the aforementioned challenges, we propose a lightweight U-shaped segmentation method, named LKMU-Lite, which integrates a Decoupled Large Kernel Attention module and multi-scale receptive field perception strategy for retinal fluid segmentation in OCT imaging. The primary contributions of this paper are summarized as follows:

1. We introduce a Decoupled Large Kernel Attention (DLKA) module that leverages the equivalent large kernel convolution receptive field to generate an attention map. This map integrates both local patterns and long-range dependencies, enhancing the salient features of the original input through a gated mechanism.
2. The Multi-scale Group Perception (MSGP) module is proposed to integrate multi-granular information by utilizing Dilated Convolutions with different receptive field scales in a grouped configuration, allowing for effective capture of features across variable lesion shapes and sizes.
3. We propose a parameter-free spatial-shift operation integrated with point-wise convolution, combined with a multi-path feature aggregation strategy, resulting in the lightweight Aggregating-Shift decoder. This innovative approach retains effective feature representation while substantially minimizing the parameters.
4. The final design of LKMU-Lite features only 1.02 M parameters. Comprehensive experiments on two OCT datasets demonstrate its state-of-the-art (SOTA) performance across multiple metrics compared to existing methods, confirming the efficiency and robustness of the proposed approach.

## 2. Related Works

UNet [10] has gained widespread adaption in medical image segmentation due to its elegant design. In this architecture, the encoder is responsible for capturing texture details and contextual features, progressively extracting meaningful semantic information as the network deepens. Simultaneously, the decoder utilizes skip connections to incrementally upsample feature maps for accurate mask prediction. Consequently, the U-shaped architecture has become a fundamental framework in numerous medical image segmentation models. For instance, Att-UNet [13] employs a gated mechanism to emphasize critical features across various spatial regions. UNet++ [12], on the other hand, enhances multi-scale feature representation by incorporating nested skip connections and establishing dense linkages between encoder and decoder layers. ResUNet++ [35] leverages attention modules alongside Atrous Spatial Pyramid Pooling, improving the fusion of information. Meanwhile, CENet [36] introduces an innovative context encoder module, designed to effectively capture and transmit contextual knowledge between the encoder and decoder components. Despite these remarkable achievements, a standard convolutional kernel in a CNN processes only a small, localized portion of the target image, leading to a restricted receptive field and limiting its capability to capture long-range dependencies. Although there are numerous attempts in the literature to address this challenge, including approaches like Dilated Convolution [18,19] and the Pyramid Pooling module [20], these methods fall short of completely solving the problem.

Leveraging the robust representation capabilities for capturing global context information inherent in self-attention mechanisms [23], ViT [22] represents a groundbreaking effort to adapt the Transformer architecture for image recognition, achieving performance comparable to state-of-the-art models. Building on this innovation, TransUNet [37] became the first to incorporate Transformer layers within a U-shaped network, enabling effective global information modeling. Extending this idea further, TransFuse [26] combines CNN and Transformer branches, leveraging their strengths to simultaneously capture global relationships and fine-grained spatial features in a compact design. Nonetheless, the self-attention mechanism in vision tasks presents a computational challenge, with complexity increasing quadratically as image resolution rises. This arises from the division of images into fixed-size patches, which are processed as sequences to enable the direct modeling of relationships between any pair of patches. To alleviate this problem, certain lightweight models have been designed, such as MedT [38], which introduces the axial attention mechanism [39] and a global–local strategy. However, the hardware requirements for training remain high.

In recent years, large kernel convolutional methods [32,33,34] have gained attention for their ability to achieve high performance while balancing computational complexity, as well as their capacity to effectively balance wide-range feature interactions and local pattern modeling. Inspired by similar concepts, many recent medical image segmentation model designs have subtly incorporated this approach. For example, ConvUNeXt [31] and CMUNeXt [40] incorporate ConvNeXt [41] principles by using larger convolution kernels to enhance feature representation capabilities, while AMSUNet [42] designs an attention module using atrous multi-scale (AMS) convolution to capture larger receptive fields, and DCSAU-Net [43] enhances model compactness and efficiency by introducing the Compact Split-Attention module and improving primary feature retention. EMCAD [44] primarily employs efficient multi-scale convolutional attention modules and a designed Large-kernel Grouped Attention Gate to create a new decoder. While these methods have achieved a certain trade-off between performance and computational cost, there is still room for further improvement in developing an even more lightweight model.

## 3. Methods

**Figure 1:**
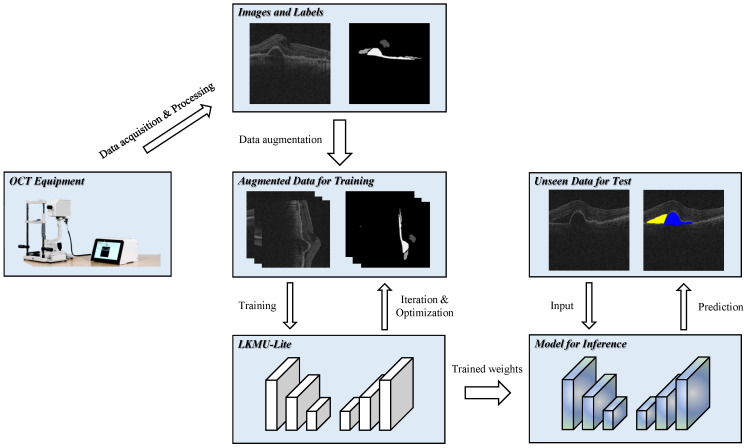
Framework of the proposed intelligent retinal fluid segmentation method.

Figure 1 illustrates the framework of the proposed intelligent retinal fluid segmentation method based on lightweight LKMU-Lite, comprising both training and testing stages. During the training phase, the parameters of LKMU-Lite are optimized for the target task. In the testing phase, the trained network predicts previously unseen data. Once trained, the network has the potential to be deployed on resource-constrained devices, enabling the efficient and accurate segmentation of retinal fluid.

The following sections will elaborate on the macrostructure and key components of LKMU-Lite.

### 3.1. Overall Architecture

**Figure 2:**
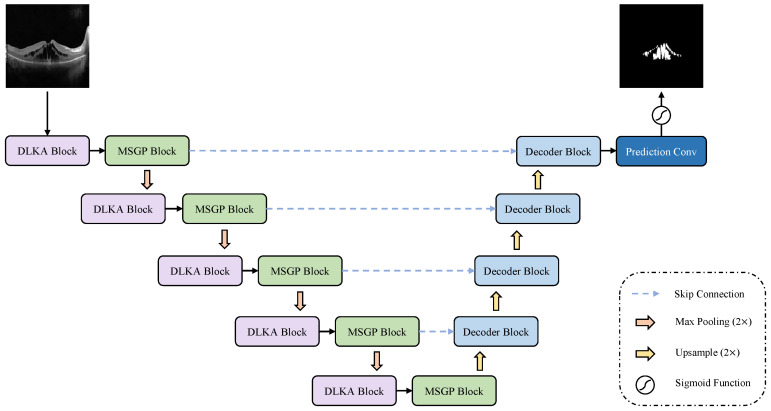
The architecture of LKMU-Lite.

The designed LKMU-Lite adopts the widely used U-shaped structure [10,11], consisting of an encoder, a decoder, and skip connections, organized into five stages, as shown in Figure 2. The number of channels in each stage is set to {32, 64, 128, 160, and 256}. In the encoder, a pooling operation is applied after each stage, except the last, to extract salient information and reduce the spatial dimensions of the feature maps. Conversely, the decoder incrementally reconstructs the output through stage-by-stage upsampling combined with convolutional modules. Skip connections link the encoder stages to their corresponding decoder stages, facilitating effective information aggregation.

Within the encoder, LKMU-Lite incorporates a Decoupled Large Kernel Attention (DLKA) module cascaded with a Multi-scale Group Perception (MSGP) module at each stage. These two modules are responsible for generating attention weights from a wide-area receptive field and for integrating features extracted from receptive fields at multiple scales, respectively. Furthermore, we propose a lightweight decoder, termed the Aggregating-Shift decoder, which combines a multi-path aggregation strategy and spatial-shift operation with point-wise convolution to achieve a balance between parameter efficiency and feature representation quality. The integration of these designs results in LKMU-Lite, which is both lightweight and high-performance.

### 3.2. Decoupled Large Kernel Attention Module

In various vision tasks, the attention mechanism functions as a selective process that emphasizes relevant, information-rich regions while suppressing irrelevant features, making it widely applicable [45]. For instance, the squeeze-excitation (SE) module [46] focuses on modeling attention at the channel level. Its variants, the CBAM [47] and BAM [48] modules, extend this capability by integrating attention across both spatial and channel dimensions. While these attention modules are well structured and easily integrated into various backbones, they rely on fixed-size convolution kernels with limited local receptive fields, making them less effective for long-range modeling. Vision self-attention mechanisms [22], on the other hand, operate as non-local processes, addressing this limitation. However, their quadratic computational complexity introduces significant overhead and high parameter counts, increasing device requirements and potentially leading to suboptimal performance in clinical scenarios with limited data.

**Figure 3:**
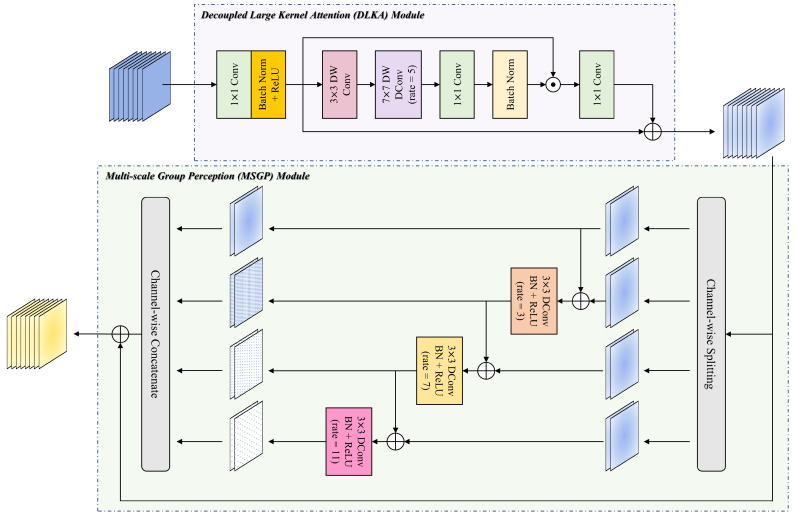
The flowchart of the Decoupled Large Kernel Attention (DLKA) module and Multi-scale Group Perception (MSGP) module. DW Conv, DWD Conv, and DConv represent Depth-wise Convolution, Depth-wise Dilated Convolution, and standard Dilated Convolution, respectively.

Building on the successful application of large kernel convolutions and their variants [32,33,34,49], we integrate the Decoupled Large Kernel Attention (DLKA) module into the encoder of LKMU-Lite, as depicted in the purple area of Figure 3. This module addresses the aforementioned limitations and achieves an optimal balance between capturing local patterns and modeling long-range dependencies.

Specifically, given the input X, a point-wise convolution is first applied to expand the channel dimensions of the feature:

(1) $$X^{\prime }=\rho \left(BN\left(PW\left(X\right)\right)\right),$$

where PW(·) denotes the point-wise convolution operation, while BN(·) and ρ(·) represent the Batch Normalization and ReLU activation function, respectively.

The core component of DLKA involves three cascaded convolutional layers, which equivalently achieve the wide receptive field of a large convolution kernel and generate the attention map Att based on this large receptive field:

(2) $$\mathrm{Att}=BN(PW(DWD_{7}^{5}\left(DW_{3}\left(X^{\prime }\right)\right))).$$

Among these three decoupled convolution operations, $DW_{3}\left(\cdot \right)$ and $DWD_{7}^{5}\left(\cdot \right)$ represent depth-wise separable convolution with a kernel size of 3×3 and depth-wise separable Dilated Convolution with a kernel size of 7×7 (rate = 5), respectively. The formula for the equivalent receptive field *R* of these two cascaded convolutional layers is given as follows:

(3) $$R=K_{1}+\left[K_{2}+\left(K_{2}-1\right)\times \left(r-1\right)\right]-1,$$

where $K_{1}$ represents the kernel size of the Depth-wise Convolution, while $K_{2}$ and *r* denote the kernel size and dilation rate of the Depth-wise Dilated Convolution, respectively. In the context of Dilated Convolution, the dilation rate defines the spacing between kernel elements during convolution operations on input data. Unlike standard convolution, where the kernel elements are contiguous, Dilated Convolution introduces gaps between kernel elements. This approach allows the receptive field of the convolutional filter to expand without increasing the number of trainable parameters or computational complexity. Consequently, it can be calculated that $DW_{3}\left(\cdot \right)$ and $DWD_{7}^{5}\left(\cdot \right)$ together capture a receptive field of size 33×33. These operations are designed to capture long-range dependencies in the spatial dimension while accounting for local patterns. Subsequently, a point-wise convolution PW(·) is applied to model features at the channel level.

Finally, the Hadamard product operation (⊙) is applied to the original feature $X^{\prime }$ and the attention map Att to emphasize important feature regions in a gating manner. This process is accompanied by a residual connection [50], which improves training convergence and enhances feature flow, resulting in the final output:

(4) $$F=PW\left(\mathrm{Att}⊙X^{\prime }\right)+X^{\prime }.$$

In addition, changes in the channel dimension at each stage of the encoder occur exclusively within the DLKA module. Specifically, the increase in channels is confined to the point-wise convolution operation in the first step of the DLKA module.

### 3.3. Multi-Scale Group Perception Module

In the retinal fluid segmentation scenario for OCT imaging, receptive field information at different scales is essential. Larger receptive fields primarily capture the overall structure of lesions and their relationship with the background, while smaller and medium-sized receptive fields focus on details of the lesion area, such as edge information. Moreover, integrating receptive field information across different scales enhances the ability to process objects of varying sizes and types. Building on this foundation, we design a lightweight Multi-scale Group Perception (MSGP) module to combine and complement the DLKA module, as illustrated in the green area of Figure 3.

After receiving the output F from the DLKA module, the feature map is split into four parts along the channel dimension. Three sub-features extract multi-scale receptive field information using Dilated Convolutions with 3×3 kernels and different dilation rates, while one sub-feature retains the original semantics. Additionally, inspired by Res2Net [51], the MSGP module incorporates adders between adjacent paths to enhance feature interaction and reuse. Finally, a concatenation operation is applied along the channel dimension to restore the feature map’s original size, followed by a residual connection to aggregate the output of the DLKA module. The above process can be represented by the following equations:

(5) $$f_{1},f_{2},f_{3},f_{4}=Split\left(F\right),$$

(6) $$k_{i}=\left\{\begin{array}{cc} f_{i} & \mathrm{if}\;\;i=1,\\ \rho \left(BN\left(D_{3}\left(f_{i}+f_{i-1}\right)\right)\right) & \mathrm{if}\;\;i=2,\\ \rho \left(BN\left(D_{7}\left(f_{i}+f_{i-1}\right)\right)\right) & \mathrm{if}\;\;i=3,\\ \rho \left(BN\left(D_{11}\left(f_{i}+f_{i-1}\right)\right)\right) & \mathrm{if}\;\;i=4, \end{array}\right.$$

(7) $$\mathrm{Out}=Concat\left(k_{1},k_{2},k_{3},k_{4}\right)+F.$$

Here, Split(·) represents the division of the input feature map along the channel dimension, and Concat(·) denotes the concatenating operation. $D_{3}\left(\cdot \right)$, $D_{7}\left(\cdot \right)$, and $D_{11}\left(\cdot \right)$ indicate standard Dilated Convolutions with dilation rates of 3, 7, and 11, respectively.

### 3.4. Lightweight Aggregating-Shift Decoder

The 3×3 convolution is commonly employed as a fundamental component of the decoder module in vanilla UNet [10] and its variations due to its effective balance between model performance and computational cost. However, the presence of skip connections introduces additional challenges: each module within a decoder stage must process feature maps from both the preceding decoder stage and the encoder branch within the same stage. As tensor channels increase due to the concatenation of multiple features, the parameters of convolution kernels also grow accordingly. Hence, this raises a natural question: how can we design a decoder with low parameters yet high representation while still preserving the capability to handle multi-channel features effectively?

**Figure 4:**
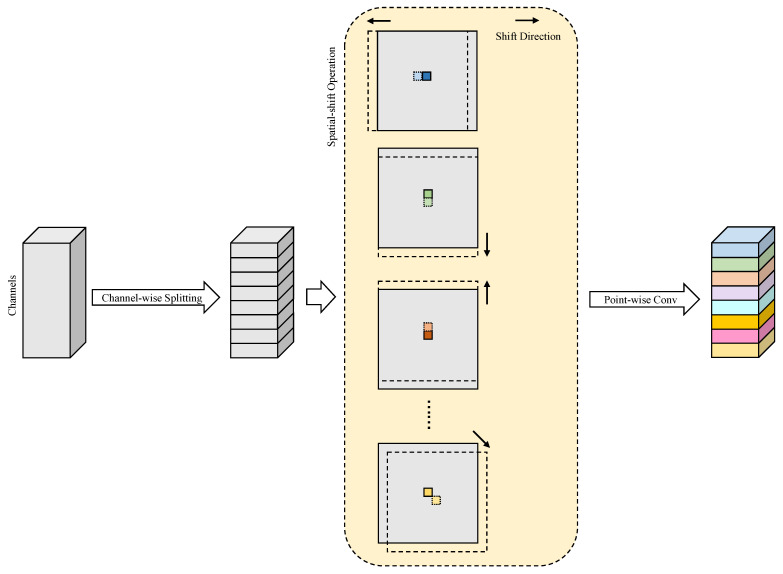
Detailed diagram of spatial-shift operation followed by point-wise convolution.

A straightforward method to reduce parameters is the use of point-wise convolution. However, this strategy limits the decoder’s ability to learn effectively due to its fixed receptive field and lack of local feature aggregation with neighboring pixels. Drawing inspiration from the spatial-shift MLP [52], we propose a parameter-free spatial-shift operation [53] combined with point-wise convolution to enable interaction among adjacent spatial pixels. As illustrated in Figure 4, the spatial-shift operation consists of two main steps: First, features are uniformly divided along the channel dimension into eight groups, and each group is shifted in a specific direction. The empty positions resulting from the shifts are padded with zeros. Subsequently, the original pixel at any given location is replaced with visual information from an adjacent pixel. Finally, a point-wise convolution is applied to extract relevant features. By utilizing eight distinct shift directions, each channel group effectively corresponds to a different neighboring pixel. When integrated with point-wise convolution, this method achieves an equivalent receptive field to a 3×3 convolution while greatly reducing both parameters and computational complexity.

**Figure 5:**
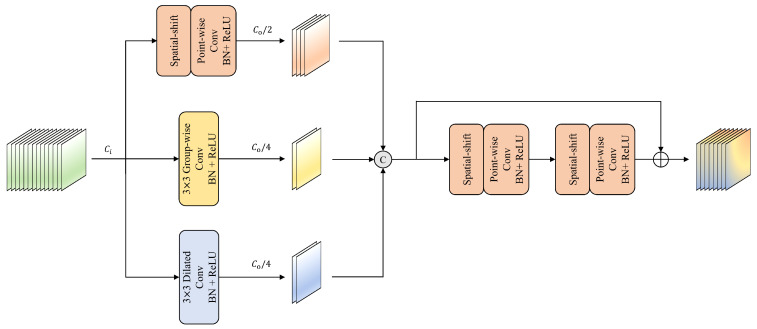
Architecture of the lightweight Aggregating-Shift decoder.

To maximize the rich semantic information from the preceding decoder and skip connection while reducing computational cost, a lightweight multi-path feature aggregation strategy is introduced for processing the concatenated feature maps. Given the input $X_{d}$ of the decoder and the output channel $C_{0}$ of the current stage, the output $Y_{d}$ of the multi-path feature aggregation module can be expressed as follows:

(8) $${\left.Y_{d}\right|}_{out=C_{0}}=Concat\left\{{\left.SPPW(X_{d})\right|}_{out=\frac{C_{0}}{2}},{\left.GC(X_{d})\right|}_{out=\frac{C_{0}}{4}}^{group=4},{\left.DC(X_{d})\right|}_{out=\frac{C_{0}}{4}}^{rate=3}\right\},$$

where SPPW(·), GC(·), and DC(·) denote the spatial-shift followed by point-wise convolution, 3×3 group-wise convolution with groups=4, and 3×3 Dilated Convolution with rate=3, respectively. The multi-path feature aggregation is followed by two spatial-shift operations combined with point-wise convolutions, integrated with a residual connection, resulting in the lightweight Aggregating-Shift decoder, as depicted in Figure 5. The final output of the decoder module is obtained as follows:

(9) $$Y=SPPW\left(SPPW(Y_{d})\right)+Y_{d}.$$

After sequential processing by the decoder at each stage, the features pass through a prediction head with a point-wise convolutional layer. The number of output channels corresponds to the number of categories.

## 4. Experiments and Discussion

### 4.1. Datasets

All experiments are conducted using two public retinal OCT datasets: Intraretinal Cystoid Fluid (ICF) [54] and the Retinal OCT Fluid Detection and Segmentation Benchmark and Challenge (RETOUCH) [55].

The ICF dataset is derived from OCT scans of patients with Diabetic Macular Edema (DME) and includes 1006 images with corresponding annotations for a single category: Cystoid Macular Edema (CME). These OCT images are meticulously annotated and selected by experts, featuring multiple CME regions with diverse shapes and sizes. Furthermore, all images in the ICF dataset have been preprocessed by the publisher, uniformly resized to 320 × 320, and their image quality enhanced using techniques such as morphological filtering, denoising, deconvolution, and contrast enhancement.

The RETOUCH dataset comprises 6936 image slices from 70 patient cases, including scans obtained from various imaging devices such as Spectralis, Cirrus, and Topcon, ensuring diversity in imaging characteristics. The image resolutions captured by the three devices vary. This dataset focuses on three primary fluid types: Intraretinal Fluid (IRF), Subretinal Fluid (SRF), and Pigment Epithelial Detachment (PED), with expert annotations provided for each. Unlike the ICF dataset, this dataset does not undergo deliberate quality enhancement steps. RETOUCH is widely used for benchmarking algorithms addressing retinal diseases like Age-related Macular Degeneration (AMD) and DME, serving as a critical resource for advancing OCT image analysis.

**Figure 6:**
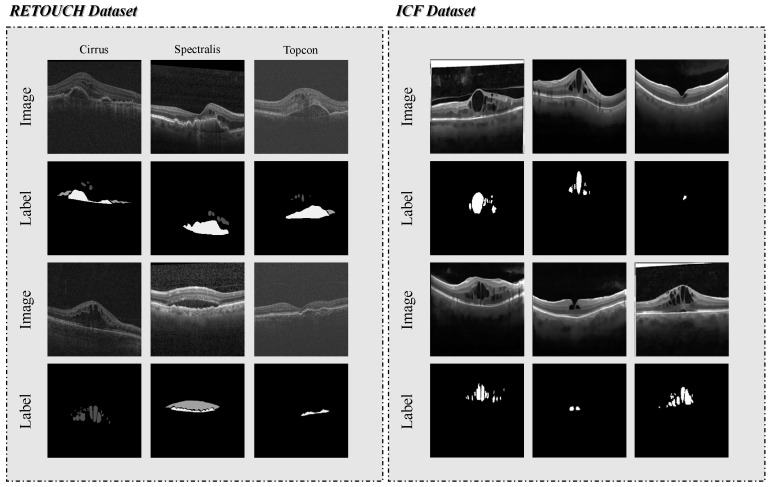
Visualizations of the RETOUCH and ICF datasets, showing both the raw images and corresponding visual labels. Compared to the ICF dataset, the RETOUCH dataset exhibits more complex scene morphology and distinct characteristics of multi-brand devices.

Visualization examples from the RETOUCH and ICF datasets are presented in Figure 6, both datasets consist of raw grayscale images. However, the more complex scene morphology and the influence of multi-brand devices make the RETOUCH dataset more challenging than the ICF dataset. Specifically, the RETOUCH dataset comprises images captured from three different devices, each exhibiting significantly different contrast and noise levels. This multi-brand nature requires addressing distributional differences between devices during model training, increasing the dataset’s complexity. Moreover, the annotated regions in the slice samples vary widely in size and display relatively complex category differences. For instance, multiple lesion categories may appear simultaneously in the same sample, or in some cases, only partially. We evaluate our method on two datasets with varying magnitudes, image quality, and classification difficulty to comprehensively assess its robustness. Each dataset is randomly partitioned into training, validation, and test sets with an 8:1:1 ratio.

### 4.2. Evaluation Metrics

The performance of the proposed LKMU-Lite on the ICF dataset is evaluated using five widely adopted metrics: Intersection over Union (IoU), Dice Similarity Coefficient (DSC), the 95th percentile Hausdorff Distance (HD95), Precision (Pre), and Sensitivity (Sen). IoU and DSC are overlap-based metrics that quantify the agreement between the predicted segmentation and the ground truth, with higher values indicating better overlap. HD95 measures the spatial distance between segmentation boundaries, emphasizing worst-case alignment errors and providing insights into boundary accuracy, where lower values signify better alignment. Precision evaluates the proportion of correctly predicted positive pixels among all predicted positives, offering an indication of the false positive rate. Sensitivity, also referred to as recall, evaluates the model’s ability to capture all true positives, highlighting its effectiveness in detecting target regions. Together, these metrics provide a comprehensive evaluation of the model’s segmentation performance, addressing region overlap, boundary precision, and classification accuracy.

For the RETOUCH dataset, IoU and DSC are used to evaluate the segmentation accuracy across three categories. Additionally, the mean IoU and mean DSC are computed to offer a comprehensive assessment of performance.

### 4.3. Implementation Details

All experiments are conducted utilizing the PyTorch framework on a workstation equipped with an Nvidia RTX 3090 GPU (24 GB). The Adam optimizer is employed, initialized with a learning rate of 1 × $10^{-3}$ and a weight decay of 1 × $10^{-4}$. Each model is trained over 150 epochs with a batch size of eight. The model training employs a polynomial learning rate decay strategy with power=0.9, where the learning rate η at each epoch is adjusted according to the following formula:

(10) $$\eta \left(\mathrm{epoch}\right)=\eta_{\mathrm{init}}{\left(1-\frac{\mathrm{epoch}}{\mathrm{num\_epochs}}\right)}^{power}.$$

This approach ensures a gradual and smooth reduction in the learning rate, promoting stable convergence and enhancing performance. The model achieving the lowest validation loss is selected for generating predictions on the test set.

All images are normalized to a standard normal distribution using the mean and standard deviation derived from the pixel distribution statistics of the ImageNet dataset and resized to a resolution of 256×256. Data augmentation techniques such as horizontal flipping, vertical flipping, and random rotation are employed. To ensure a fair comparison, all experiments are conducted under consistent settings, with all models trained from scratch.

The loss function of our method is a composite of Binary Cross-entropy (BCE) loss and Dice Loss. Cross-entropy is used to measure the difference between two probability distributions and encode the average amount of information required for the real distribution based on the predicted distribution. BCE Loss minimizes prediction uncertainty, enabling the model to concentrate the output probability distribution on the true category as much as possible. However, it primarily focuses on pixel-level classification and is sensitive to imbalanced category ratios. Dice Loss, on the other hand, plays a complementary role by directly optimizing the overlap between the target and predicted areas. It is more robust to category imbalances and is particularly effective for segmenting small and medium-sized targets. The descriptions of these two losses are as follows:

(11) $$L_{BCE}\left(P,G\right)=-\frac{1}{N}\;\sum_{i=1}^{N}\;\left[g_{i}\;\cdot \;\log\left(p_{i}\right)\;+\;\left(1\;-\;g_{i}\right)\;\cdot \;\log\left(1\;-\;p_{i}\right)\right],$$

(12) $$L_{Dice}\left(P,G\right)=1-\left(2\sum_{i=1}^{N}p_{i}\;\cdot \;g_{i}+\epsilon \right)/\left(\sum_{i=1}^{N}p_{i}^{2}+\sum_{i=1}^{N}g_{i}^{2}+\epsilon \right),$$

where *P* represents the predicted segmentation, and *G* denotes the ground truth. Here, $p_{i}$ and $g_{i}$ represent individual pixels in *P* and *G*, respectively. The total number of pixels is denoted by *N*, and ϵ serves as a smoothing term. With the specific weights often determined based on empirical experience, the comprehensive loss function is expressed as follows:

(13) $$L=0.5\cdot L_{BCE}\left(P,G\right)+L_{Dice}\left(P,G\right).$$

### 4.4. Comparison with State-of-the-Art Models

We compare the proposed LKMU-Lite with a series of state-of-the-art medical image segmentation models. Specifically, the compared methods include UNet [10] and its common variants [12,13,35,36], segmentation models utilizing Vision Transformers [26,37,38], and several methods proposed within the past two years [31,40,42,43,44].

Table 1 and Table 2 show the quantitative results of various baselines on the ICF and RETOUCH datasets, respectively. Furthermore, to assess efficiency, we present the parameters (Params) and computational complexity of LKMU-Lite alongside other methods in Table 1. The model complexity is evaluated based on Floating-Point Operations (FLOPs), with both FLOPs and parameters determined using an input image of size 256×256 and a batch size of one. According to the results, LKMU-Lite outperforms all compared methods across every metric on the ICF dataset. The RETOUCH dataset presents a greater challenge due to the lack of preprocessing to enhance image quality, its complex scene morphology, and the influence of multi-brand devices inherent to the task. However, apart from the IoU score for SRF segmentation, which is slightly lower than that of TransFuse-S, LKMU-Lite still achieves the most competitive results across other metrics. Notably, in the IRF and PED categories, LKMU-Lite demonstrates a significant performance advantage, resulting in the best mean IoU and DSC scores.

**Figure 7:**
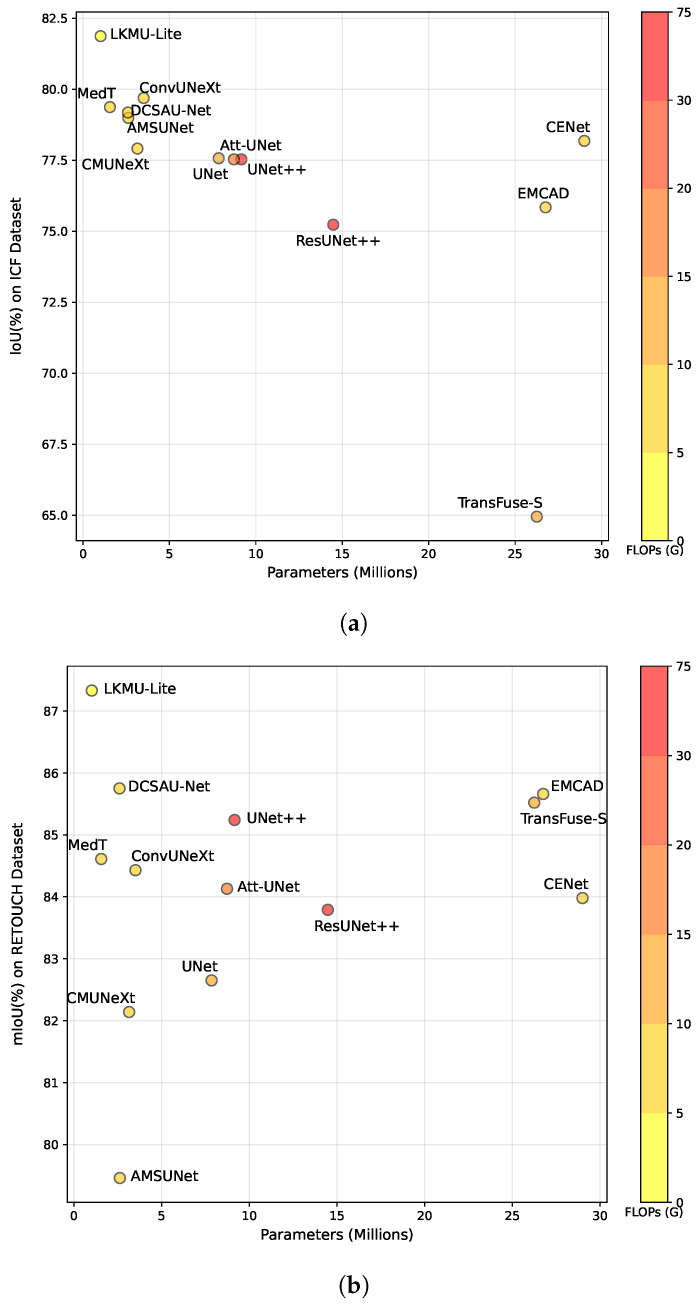
Scatter plots comparing various methods based on parameters, computational complexity, and mean IoU (mIoU) performance on (**a**) the ICF dataset and (**b**) the RETOUCH dataset. Note that TransUNet, which has significantly higher parameters, is excluded to provide a clearer visualization of the performance differences among the compared models.

In addition to its performance advantages, LKMU-Lite is highly efficient in terms of computing resources. For instance, compared to the vanilla UNet, our model has 6.7 times fewer parameters and 2.67 times lower computational complexity. While the Transformer-based TransUNet, which features a global receptive field modeling capability, achieves competitive performance on both datasets, it comes at the cost of requiring 102.25 times more parameters and 9.09 times higher complexity compared to LKMU-Lite. According to the scatter plot in Figure 7, scatter points closer to the upper left corner indicate a better balance between efficiency and performance. This suggests that LKMU-Lite achieves an optimal trade-off between these two factors.

**Figure 8:**
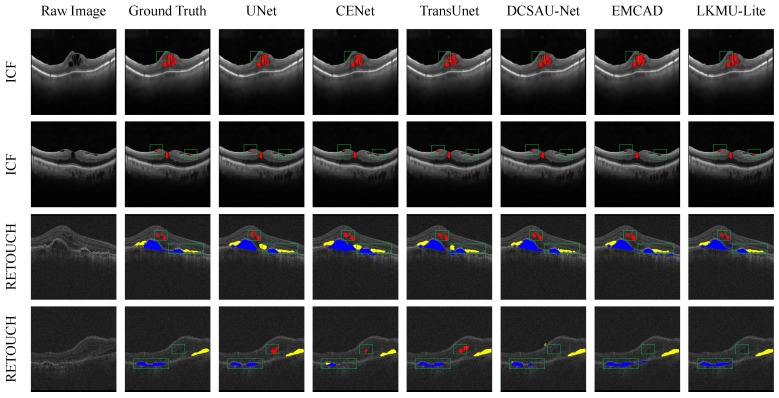
Comparison of visualized predictions between LKMU-Lite and selected baseline models on the test sets of the ICF and RETOUCH datasets. The red color represents Intraretinal Fluid (IRF), while the Cystoid Macular Edema (CME) in the ICF dataset is a specific form of IRF. The yellow and blue colors represent Subretinal Fluid (SRF) and Pigment Epithelial Detachment (PED), respectively. The areas highlighted by green boxes emphasize the improvements of our method over other models in mitigating mis-segmentation and missed segmentation issues.

Figure 8 presents a comparative analysis of segmentation visualizations, with predicted regions overlaid using non-transparent colors on the original image to represent different target categories. Other methods exhibit more evident mis-segmentation or even missed segmentation in the slice examples shown in the figure. In contrast, LKMU-Lite, leveraging the combination of the large kernel attention mechanism and multi-scale receptive field integration, provides a more accurate depiction of retinal fluid areas of varying shapes and sizes.

### 4.5. Ablation Analysis

To validate the effectiveness of the individual components in LKMU-Lite, we conduct ablation studies on both the ICF and RETOUCH datasets. The experimental configurations are categorized into three groups as follows:

(1) To determine the most suitable equivalent Receptive Field Size for the DLKA module, which is primarily controlled by the Depth-wise Dilated Convolution (DWD Conv) kernel size, we evaluate four models with different configurations: A (DWD Conv with a 3×3 kernel), B (DWD Conv with a 5×5 kernel), C (LKMU-Lite), and D (DWD Conv with a 9×9 kernel).
(2) The effectiveness of the proposed DLKA and MSGP modules are evaluated, respectively. Specifically, the DLKA module is replaced with a 3×3 convolutional block from VGG [17], while the MSGP module remains unchanged, resulting in a model named 3×3 Conv + MSGP. Conversely, when the MSGP module is replaced with a 3×3 convolutional block, the modified model is denoted as DLKA + 3×3 Conv. Additionally, replacing both the DLKA and MSGP modules with 3×3 convolutional blocks results in an encoder backbone similar to that of a vanilla UNet [10], and the modified model is named 3×3 Conv + 3×3 Conv.
(3) To evaluate the proposed Aggregating-Shift decoder, we introduce two variants of the decoder module: one composed entirely of 3×3 convolutions, forming a standard convolutional decoder with a residual connection, and another that replaces all spatial-shift point-wise convolutions with 3×3 convolutions while retaining the multi-path feature aggregation structure. The modified models are named 3×3 Conv Decoder and 3×3 Conv Decoder-mod, respectively.

The quantitative results under different equivalent receptive field settings are presented in Table 3 and Table 4. The optimal DWD Conv kernel size is 7×7 rather than 9×9. The experimental results indicate that an excessively large receptive field is not necessary for achieving good segmentation performance. This is because increasing the receptive field too much may introduce unnecessary features, leading to information redundancy and negatively impacting the model’s performance when handling lesions with rich local details. Therefore, at the feature modeling level, a balance between wide-range dependencies and local patterns should be considered.

The analysis of the effectiveness of the DLKA and MSGP modules is shown in Table 5 and Table 6. For 3×3 Conv + 3×3 Conv, which stacks the encoder using a fixed-size kernel convolution, the performance is similar to that of UNet, as shown in Table 1 and Table 2. This further demonstrates that the limited receptive field capture range struggles to adequately address the variable lesion feature patterns in OCT imaging. On this basis, the introduction of either the DLKA or MSGP modules not only improves performance but also helps reduce parameters and computational complexity. Furthermore, the combination of the large kernel attention mechanism (DLKA) and the multi-scale receptive field integration strategy (MSGP) demonstrates the most competitive performance.

For the evaluation of the decoder, by comparing the Aggregating-Shift decoder with the 3×3 Conv decoder-mod from Table 7 and Table 8, it is evident that the spatial-shift combined with point-wise convolution significantly reduces computational overhead, specifically lowering parameters by approximately 55% and complexity by around 62%, without sacrificing feature representation. Additionally, there is a significant performance gap between the pure 3×3 Conv decoder and the modified 3×3 Conv decoder-mod. This demonstrates that introducing the multi-path feature aggregation method enriches the gradient return path without increasing overhead, which is beneficial for accurate segmentation.

## 5. Conclusions

In this paper, we introduce LKMU-Lite, a lightweight U-shaped segmentation model specifically designed for retinal fluid segmentation. Concretely, we incorporate a Decoupled Large Kernel Attention (DLKA) module to capture both local patterns and long-range dependencies, enhancing feature representation through a gated mechanism. Additionally, a Multi-scale Group Perception (MSGP) module is designed to capture multi-granular features using Dilated Convolutions at varying receptive field scales, enabling the effective segmentation of different lesion shapes and sizes. Finally, the lightweight Aggregating-Shift decoder reduces computational overhead while preserving segmentation accuracy by combining spatial-shift operations and point-wise convolutions. Extensive experiments on the ICF and RETOUCH datasets demonstrate the superiority of LKMU-Lite over existing models while requiring significantly fewer parameters and maintaining low complexity. Additionally, LKMU-Lite holds potential for application in medical image segmentation tasks across other imaging modalities. In future works, we will integrate cross-validation strategies to further explore the performance of LKMU-Lite in other tasks and make appropriate improvements to develop a more real-time and versatile segmentation architecture.

## Figures and Tables

**Table 1:** Quantitative evaluation results on the ICF dataset compared to existing SOTA methods. Parameters and computational complexity for each model are also provided. The symbol “↑” indicates that higher values are preferred, whereas “↓” indicates that lower values are better. The best results are highlighted in bold.

Model	Year	Metrics					Resource Usage	
		IoU (%) ↑	DSC (%) ↑	HD95 ↓	Pre (%) ↑	Sen (%) ↑	Params ↓	FLOPs ↓
UNet [10]	2015	77.57	86.90	3.64	88.82	86.72	7.85 M	14.01 G
UNet++ [12]	2018	77.53	86.81	4.50	89.55	85.98	9.16 M	34.66 G
Att-UNet [13]	2018	77.53	86.92	4.73	89.36	86.18	8.73 M	16.76 G
CENet [36]	2019	78.18	87.31	3.74	89.32	86.66	29.00 M	7.16 G
ResUNet++ [35]	2019	75.23	85.13	4.46	87.07	85.21	14.48 M	70.77 G
TransUNet [37]	2021	78.87	87.68	4.39	89.19	87.49	105.32 M	38.53 G
TransFuse-S [26]	2021	64.95	76.23	9.43	82.20	73.41	26.25 M	11.53 G
MedT [38]	2021	79.37	88.12	3.94	89.94	87.45	1.56 M	5.62 G
ConvUNeXt [31]	2022	79.69	88.26	2.66	90.69	87.34	3.51 M	7.18 G
AMSUNet [42]	2023	78.99	87.87	3.60	89.51	87.73	2.62 M	6.06 G
DCSAU-Net [43]	2023	79.18	87.96	4.05	89.43	88.02	2.60 M	6.72 G
CMUNeXt [40]	2024	77.91	87.12	2.95	90.16	85.84	3.15 M	7.32 G
EMCAD [44]	2024	75.84	85.79	3.38	87.13	85.80	26.76 M	5.83 G
LKMU-Lite (ours)	2024	**81.87**	**89.66**	**2.11**	**91.13**	**89.16**	**1.02 M**	**3.82 G**

**Table 2:** Quantitative evaluation results on the RETOUCH dataset. The best results are highlighted in bold.

Model	Year	IoU (%) ↑				DSC (%) ↑			
		IRF	SRF	PED	Mean	IRF	SRF	PED	Mean
UNet [10]	2015	80.31	90.32	77.33	82.65	82.86	91.89	76.62	83.79
UNet++ [12]	2018	80.28	89.47	85.97	85.24	82.81	90.91	87.20	86.97
Att-UNet [13]	2018	79.05	88.57	84.76	84.13	81.81	89.46	85.96	85.74
CENet [36]	2019	78.12	89.33	84.50	83.98	81.05	91.02	85.92	86.00
ResUNet++ [35]	2019	79.46	89.58	82.33	83.79	81.98	90.88	83.15	85.34
TransUNet [37]	2021	79.84	90.90	87.42	86.05	82.57	92.22	88.89	87.89
TransFuse-S [26]	2021	79.07	**91.08**	86.42	85.52	81.85	92.72	88.05	87.54
MedT [38]	2021	78.96	89.66	85.21	84.61	81.76	91.06	86.60	86.47
ConvUNeXt [31]	2022	78.72	89.16	85.42	84.43	81.46	90.28	86.66	86.13
AMSUNet [42]	2023	75.10	80.72	82.56	79.46	76.96	80.52	83.80	80.43
DCSAU-Net [43]	2023	80.19	90.29	86.77	85.75	83.01	91.60	88.57	87.73
CMUNeXt [40]	2024	77.89	83.91	84.61	82.14	80.77	84.69	85.68	83.71
EMCAD [44]	2024	79.54	90.85	86.59	85.66	82.30	92.24	87.59	87.38
LKMU-Lite (ours)	2024	**81.97**	90.98	**89.04**	**87.33**	**85.31**	**92.78**	**91.15**	**89.75**

**Table 3:** Evaluation on the ICF dataset under different equivalent receptive field settings, where “RF size” stands for Receptive Field Size.

Settings	DW Kernel	DWD Kernel	RF Size	Metrics				
				IoU (%) ↑	DSC (%) ↑	HD95 ↓	Pre (%) ↑	Sen (%) ↑
A	3×3	3×3	13×13	81.61	89.54	2.64	89.76	**90.13**
B	3×3	5×5	23×23	80.17	88.59	**2.01**	90.10	87.99
C (LKMU-Lite)	3×3	7×7	33×33	**81.87**	**89.66**	2.11	**91.13**	89.16
D	3×3	9×9	43×43	79.84	88.44	2.50	89.20	88.76

**Table 4:** Evaluation on the RETOUCH dataset under different equivalent receptive field settings.

Settings	DW Kernel	DWD Kernel	RF Size	IoU (%) ↑/DSC (%) ↑			
				IRF	SRF	PED	Mean
A	3×3	3×3	13×13	80.84/84.14	90.88/92.63	87.82/89.78	86.51/88.85
B	3×3	5×5	23×23	81.46/84.75	91.20/92.93	87.41/89.16	86.69/88.95
C (LKMU-Lite)	3×3	7×7	33×33	**81.97**/**85.31**	90.98/92.78	**89.04**/**91.15**	**87.33**/**89.75**
D	3×3	9×9	43×43	81.10/84.30	**91.64**/**93.37**	88.44/90.12	87.06/89.26

**Table 5:** Ablation study of the DLKA and MSGP modules on the ICF dataset.

Settings	Metrics					Resource Usage	
	IoU (%) ↑	DSC (%) ↑	HD95 ↓	Pre (%) ↑	Sen (%) ↑	Params ↓	FLOPs ↓
3×3 Conv + 3×3 Conv	79.24	87.92	3.68	89.99	87.20	2.15 M	5.73 G
3×3 Conv + MSGP	81.29	89.37	2.57	**91.17**	88.62	1.33 M	3.94 G
DLKA + 3×3 Conv	80.22	88.68	**2.03**	90.70	87.79	1.85 M	5.61 G
DLKA + MSGP (LKMU-Lite)	**81.87**	**89.66**	2.11	91.13	**89.16**	**1.02 M**	**3.82 G**

**Table 6:** Ablation study of the DLKA and MSGP modules on the RETOUCH dataset.

Settings	IoU (%) ↑				DSC (%) ↑			
	IRF	SRF	PED	Mean	IRF	SRF	PED	Mean
3×3 Conv + 3×3 Conv	80.19	**91.14**	76.36	82.56	82.93	92.49	75.73	83.72
3×3 Conv + MSGP	80.67	90.93	87.71	86.44	83.94	92.27	89.36	88.52
DLKA + 3×3 Conv	80.82	90.83	87.91	86.52	83.81	92.58	89.88	88.76
DLKA + MSGP (LKMU-Lite)	**81.97**	90.98	**89.04**	**87.33**	**85.31**	**92.78**	**91.15**	**89.75**

**Table 7:** Ablation study of the Aggregating-Shift decoder on the ICF dataset.

Settings	Metrics					Resource Usage	
	IoU (%) ↑	DSC (%) ↑	HD95 ↓	Pre (%) ↑	Sen (%) ↑	Params ↓	FLOPs ↓
3×3 Conv decoder	79.85	88.45	2.67	89.71	88.28	2.45 M	10.99 G
3×3 Conv decoder-mod	81.22	89.24	2.48	**91.77**	88.14	2.25 M	9.95 G
Aggregating-Shift decoder	**81.87**	**89.66**	**2.11**	91.13	**89.16**	**1.02 M**	**3.82 G**

**Table 8:** Ablation study of the Aggregating-Shift decoder on the RETOUCH dataset.

Settings	IoU (%) ↑				DSC (%) ↑			
	IRF	SRF	PED	Mean	IRF	SRF	PED	Mean
3×3 Conv decoder	77.48	87.49	84.69	83.22	79.94	88.76	85.33	84.68
3×3 Conv decoder-mod	81.26	90.42	88.15	86.61	84.51	91.92	89.80	88.74
Aggregating-Shift decoder	**81.97**	**90.98**	**89.04**	**87.33**	**85.31**	**92.78**	**91.15**	**89.75**

## Data Availability

The data can be accessed upon reasonable request to the corresponding authors.

## Abbreviations

The following abbreviations are used in this manuscript:

OCT	Optical Coherence Tomography
AI	Artificial Intelligence
FCN	Fully Convolutional Network
CNN	Convolutional Neural Network
ViT	Vision Transformer
NLP	Natural Language Processing
DLKA	Decoupled Large Kernel Attention
MSGP	Multi-scale Group Perception
SOTA	state-of-the-art
SE	squeeze-excitation
DW Conv	Depth-wise Convolution
DWD Conv	Depth-wise Dilated Convolution
DConv	Dilated Convolution
ICF	Intraretinal Cystoid Fluid
RETOUCH	Retinal OCT Fluid Detection and Segmentation Benchmark and Challenge
DME	Diabetic Macular Edema
CME	Cystoid Macular Edema
IRF	Intraretinal Fluid
SRF	Subretinal Fluid
PED	Pigment Epithelial Detachment
AMD	Age-related Macular Degeneration
IoU	Intersection over Union
DSC	Dice Similarity Coefficient
HD95	95th percentile Hausdorff Distance
Pre	Precision
Sen	Sensitivity
BCE	Binary Cross-entropy
Params	parameters
FLOPs	Floating-Point Operations
mIoU	mean Intersection over Union
RF Size	Receptive Field Size
